# Safety and immunogenicity of concomitant quadrivalent meningococcus ACYW-TT and meningococcus group B vaccines

**DOI:** 10.1038/s41541-026-01508-7

**Published:** 2026-07-01

**Authors:** Monica A. McArthur, Adam Finn, Lawrence Barnes, Richard Burkimsher, Katrina Cathie, Eva Galiza, Paul Heaton, Nicholas Jacobsen, Stephen Jones, Sian Ludman, Patrick Moore, Clare Murray, Daniel Owens, Alexander Trotman, Ray Borrow, Julie Chaix, Arlette Delgado, Estelle Dubois, Youjun Huang, Olga Syrkina, Betzana Zambrano, Mandeep Singh Dhingra, Christine Rehm

**Affiliations:** 1 Global Clinical Development Strategy, Sanofi, Swiftwater, PA USA; 2 Bristol Vaccine Centre, Schools of Population Health Sciences and Cellular and Molecular Medicine, University of Bristol and Bristol Royal Hospital for Children, University Hospitals Bristol NHS Foundation Trust, Bristol, UK; 3 Rame Medical Limited, Penntorr Health, Torpoint, Cornwall, UK; 4 Mounts Bay Medical Ltd, Connor Downs Surgery, Connor Downs, Hayle, Cornwall, UK; 5 National Institute for Health Research Southampton Clinical Research Facility and Biomedical Research Centre, University Hospital Southampton NHS Foundation Trust, and Faculty of Medicine and Institute for Life Sciences, University of Southampton, Southampton, UK; 6 Vaccine Institute, City St George’s, University of London, London, UK; 7 Department of Paediatrics, Yeovil District Hospital, Yeovil, Somerset, UK; 8 Newquay Health Centre, Newquay, Cornwall, UK; 9 Royal United Hospital, Combe Park, Bath, Avon, UK; 10 Royal Devon University Hospital, Exeter, UK; 11 The Adam Practice, Poole, Dorset, UK; 12 Royal Manchester Children’s Hospital, Manchester University NHS Foundation Trust, and Division of Immunology, Immunity to Infection and Respiratory Medicine, University of Manchester, Manchester, UK; 13 Chaddlewood Surgery, Plympton, Plymouth, UK; 14 Vaccine Evaluation Unit, UK Health Security Agency, Manchester, UK; 15 Global Clinical Immunology, Sanofi, Marcy l’Etoile, France; 16 Global Pharmacovigilance-Vaccines, IQVIA; and Patient Safety & Pharmacovigilance, Sanofi, Madrid, Spain; 17 Global Clinical Operations, Sanofi, Marcy l’Etoile, France; 18 Evidence Generation and Decision Sciences, Sanofi, Chengdu, China; 19 Patient Safety and Pharmacovigilance, Sanofi R&D, Cambridge, MA USA; 20 Global Clinical Development Strategy, Sanofi, Montevideo, Uruguay

**Keywords:** Diseases, Health care, Immunology, Medical research, Microbiology

## Abstract

Infants are vulnerable to meningococcal disease. In this open-label phase III study (NCT03632720; registered 13/8/2018), infants were randomised to quadrivalent ACWY conjugated vaccine, MenACYW-TT, at 3 and 12–13 months and four-component meningococcus group B vaccine (4CMenB) at 2, 4, and 12–13 months (Group 1), MenACYW-TT at 3 and 12–13 months and 4CMenB at 2 and 4 months (Group 2), or 4CMenB at 2, 4, and 12–13 months (Group 3). Routine vaccines were also administered per UK schedule. Primary outcome of non-inferiority of seroprotection rates against the four capsular groups (A, C, W, and Y) in Group 1 versus Group 2 at day 30 after the second vaccine dose was demonstrated with seroprotection rates ≥99% against all capsular groups in both groups. In Group 3, cross-reactivity was observed, with hSBA titres against capsular groups A and C increasing >10-fold after the third 4CMenB dose. Reactogenicity was consistent with the known profiles of both vaccines; one case of anaphylaxis related to MenACYW-TT was reported and resolved without sequelae. No new safety concerns were identified.

## Introduction

Invasive meningococcal disease (IMD), which commonly manifests as sepsis and/or meningitis, is a life-threatening illness caused by infection with the bacterium *Neisseria meningitidis*. Vaccination is central in the prevention of IMD. Implementation of meningococcal vaccination programmes for children has been instrumental in the decline in IMD rates^1,2^.

In the UK, the introduction of the meningococcus capsular group C (MenC) vaccine into the routine immunisation schedule at ages 2, 3, and 4 months in November 1999, as well as school-based programmes, led to over 95% reduction in MenC disease rates over the subsequent 18 years (2000–2018)^3–5^. The emergence of meningococcus capsular group W (MenW) and the continued predominance of meningococcus capsular group B (MenB) led to the introduction of the quadrivalent ACWY conjugated vaccine (MenACWY) for adolescents and the MenB vaccine into the national infant immunization schedule in 2015^6^. Subsequently, the MenB vaccination programme led to a 75% reduction in the incidence of MenB disease in the age group eligible for the vaccine during the first three years of the programme in England^7^. In addition, the MenACWY vaccination programme led to reductions in carriage of MenW and MenY by 73% and 69%, respectively, in the three years after introduction and sustained low MenC carriage in UK^8^.

The MenACYW-TT vaccine (tetanus toxoid-conjugated; MenQuadfi®, Sanofi, Swiftwater, PA, USA) was developed for the immunisation of individuals against IMD across a broad age range. At the time of this study, MenACYW-TT was licensed for use in the EU and other countries as a single dose in individuals aged 12 months and older, and in the USA as single dose in those aged 2 years and older. Recently, in the USA, the age indication was extended down to 6 weeks of age based on studies demonstrating broad protection in this most vulnerable age group^9–11^. To complement these efforts, this phase III study (MET52, NCT03632720) was undertaken to evaluate the safety and immunogenicity after the second dose of MenACYW-TT as a two-dose schedule (3 months and 12–13 months of age) in infants and toddlers when co-administered with the four-component meningococcus capsular group B vaccine (4CMenB) in the UK. Despite the UK’s adolescent-targeted conjugate ACWY vaccination strategy—which relies on indirect effects on carriage and transmission to confer protection across age groups—European immunisation programmes remain markedly heterogeneous, with several countries extending vaccination to infants and/or toddlers and variably incorporating 4CMenB. In this context, the results of the present study retain broad and continuing relevance to assist in programme-level immunisation practices. The primary objective of the current study was to demonstrate the non-inferiority of the antibody responses to meningococcus capsular groups A, C, W, and Y in terms of seroprotection (serum bactericidal assay using human complement [hSBA] antibody titres ≥1:8) when MenACYW-TT was administered concomitantly with 4CMenB in the second year of life compared to when MenACYW-TT was given alone. Non-inferiority was pre-defined as being present if the lower limits of the two-sided 95% confidence intervals (CIs) of the difference between the two percentages were above –10% for all four capsular groups.

## Results

### Participants

**Fig. 1:**
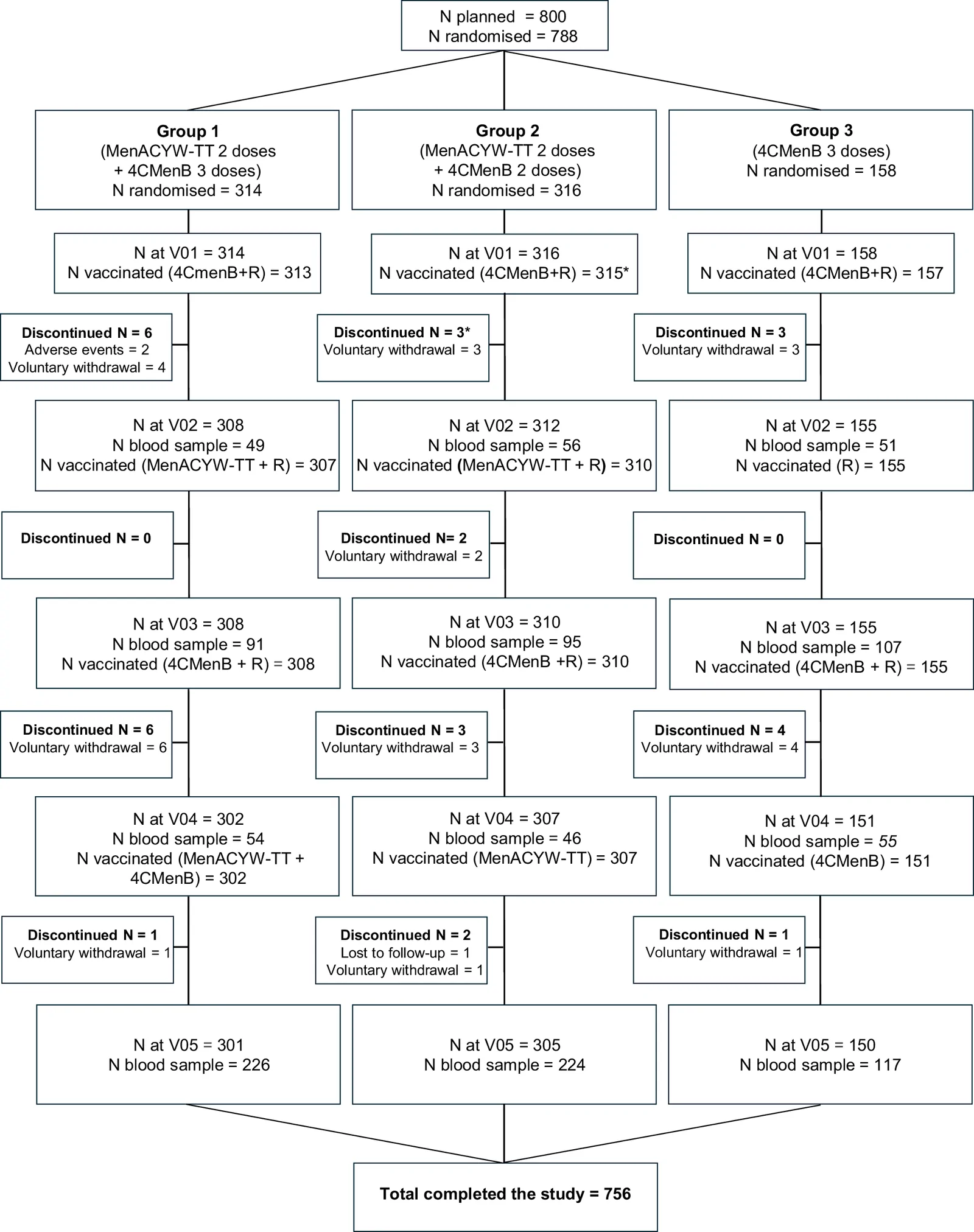
Flow of participants through the study. Flow diagram showing participant enrollment, randomisation, and study visits and procedures. A total of 788 participants were randomised 2:2:1 to Group 1 (*N* = 314), Group 2 (*N* = 316), and Group 3 (*N* = 158). A total of 756 participants completed the study. *One participant in Group 2 was a screen failure and thus did not receive any study vaccine at Visit 1. No demographic data were collected for this participant. This participant was not included as discontinued after Visit 1. Infants in Group 2 received their third dose of 4CMenB after completion of the last study visit procedures. 4CMenB four-component meningococcus capsular group B vaccine, MenACYW-TT quadrivalent meningococcus ACYW tetanus toxoid-conjugate vaccine, R routine pediatric vaccines, V visit.

The study was undertaken between 10 October 2018 and 5 December 2022, with 788 participants enrolled and randomised 2:2:1 to receive MenACYW-TT at 3 months and 12–13 months of age and 4CMenB (Bexsero®, GlaxoSmithKline, Rixensart, Belgium) at 2, 4, and 12–13 months of age (Group 1), MenACYW-TT at 3 months and 12–13 months of age and 4CMenB at 2 and 4 months of age (Group 2) (the booster dose of 4CMenB being given after the end of the study), or 4CMenB at 2, 4, and 12–13 months of age (Group 3). The participants’ flow through the study is summarised in Fig. 1. Of those enrolled, 785 received at least one study injection and had associated safety data available, and these comprised the safety analysis set (SafAS). A total of 756 completed the study. Discontinuations from the study were mainly due to voluntary withdrawals (*n* = 28), and two were after adverse events, both of which were considered unrelated to study vaccines. The demographic characteristics of the participants are summarised in Table 1.

**Table 1:** Summary of participants’ demographic characteristics at baseline (all randomised participants)

	Group 1	Group 2	Group 3	All
	(*N* = 314)	(*N* = 316)	(*N* = 158)	(*N* = 788)
Sex: *n* (%)				
Male	166 (52.9)	161 (50.9)	77 (48.7)	404 (51.3)
Female	148 (47.1)	154 (48.7)	81 (51.3)	383 (48.6)
Missing	0	1 (0.3)	0	1 (0.1)
Age (Days)				
*M*	314	315	158	787
Mean (SD)	64.7 (6.88)	63.9 (6.73)	64.8 (7.03)	64.4 (6.85)
Min; Max	56.0; 85.0	56.0; 89.0	56.0; 88.0	56.0; 89.0
Median	63.0	62.0	63.0	63.0
Q1; Q3	60.0; 68.0	59.0; 67.0	60.0; 68.0	59.0; 68.0
Racial origin: *n* (%)				
White	300 (95.5)	296 (93.7)	148 (93.7)	744 (94.4)
Asian	1 (0.3)	4 (1.3)	4 (2.5)	9 (1.1)
Black	0	2 (0.6)	0	2 (0.3)
Multiple Origin	4 (1.3)	10 (3.2)	4 (2.5)	18 (2.3)
Unknown	9 (2.9)	3 (0.9)	2 (1.3)	14 (1.8)
Missing	0	1 (0.3)	0	1 (0.1)
Ethnicity: *n* (%)				
Hispanic/Latino	4 (1.3)	5 (1.6)	1 (0.6)	10 (1.3)
Not Hispanic/Latino	307 (97.8)	307 (97.2)	154 (97.5)	768 (97.5)
Not Reported	1 (0.3)	2 (0.6)	1 (0.6)	4 (0.5)
Unknown	2 (0.6)	1 (0.3)	2 (1.3)	5 (0.6)
Missing	0	1 (0.3)	0	1 (0.1)

Group 1: MenACYW-TT at 3 months and at 12–13 months of age; 4CMenB at 2, 4, and 12–13 months of age.

Group 2: MenACYW-TT at 3 months and at 12–13 months of age; 4CMenB at 2 and 4 months of age, with the third dose of 4CMenB administered after completion of the last study visit procedures.

Group 3: 4CMenB at 2, 4, and 12–13 months of age.

*4CMenB* four-component meningococcus capsular group B vaccine, *M* number of participants with available data for the relevant endpoint, *MenACYW-TT* quadrivalent meningococcus ACYW tetanus toxoid-conjugate vaccine, *n* number of participants fulfilling the item listed in the first column, *Q1* first quartile, *Q3* third quartile, *SD* standard deviation.

The per-protocol analysis set (PPAS) served as the analysis set for immunogenicity. The PPAS was divided into three sets depending on whether participants were required or selected to provide a blood sample, had no relevant protocol deviations and had valid post-vaccination serology (after the first vaccination, and before and after the second vaccination). It included: 78 and 77 participants in Groups 1 and 2, respectively, at D30 after vaccination at 3 months of age; 41 and 36 participants, respectively, at 12–13 months of age; and 178 and 169, respectively, at D30 after vaccination at 12–13 months of age. The PPAS numbers in Group 3 at the same time points were 84, 42, and 86, respectively.

A total of 145 (18.5%) participants were affected by disruptions relating to coronavirus disease 2019 (COVID-19), including those with at least one major or critical protocol deviation or who were unable to complete the study due to pandemic-related restrictions. Since this number exceeded the pre-planned 10% threshold, a sensitivity analysis was performed to summarise the main immunogenicity and safety endpoints for these participants.

### Immunogenicity

The seroprotection rates (proportion with hSBA titres ≥1:8) against the four meningococcus capsular groups (A, C, W, and Y) 30 days after the second vaccination at 12–13 months of age were ≥99% when MenACYW-TT was administered concomitantly with 4CMenB (Group 1) and when it was administered alone (Group 2) (Table 2). The lower limits of the 95% CIs for the differences between Groups 1 and 2 in seroprotection rates against all four meningococcus capsular groups were above the pre-defined threshold of −10% (Table 2). Thus, MenACYW-TT administered concomitantly with 4CMenB demonstrated non-inferiority to MenACYW-TT administered alone in terms of seroprotection against all four MenACYW-TT vaccine capsular groups.

**Table 2:** Non-inferiority of hSBA vaccine seroprotection (titre ≥1:8) at D30 after the second MenACYW-TT vaccination administered at 12–13 months of age for Group 1 versus Group 2 (PPAS at D30 after vaccination at 12–13 months of age)^a^

Capsular group	Group 1 (*N* = 178)			Group 2 (*N* = 169)				Group 1 – Group 2		
	*n*/*M*	%	(95% CI)	*n*/*M*	%	(95% CI)	Difference %		(95% CI)	Non-inferiority
A	159/159	100	(97.7;100)	155/156	99.4	(96.5;100)	0.64		(–1.78;3.54)	Yes
C	168/168	100	(97.8;100)	165/165	100	(97.8;100)	0.00		(–2.24;2.28)	Yes
Y	161/161	100	(97.7;100)	159/159	100	(97.7;100)	0.00		(–2.33;2.36)	Yes
W	171/172	99.4	(96.8;100)	160/160	100	(97.7;100)	–0.58		(–3.22;1.81)	Yes

Group 1: MenACYW-TT at 3 months and at 12–13 months of age; 4CMenB at 2, 4, and 12–13 months of age.

Group 2: MenACYW-TT at 3 months and at 12–13 months of age; 4CMenB at 2 and 4 months of age, with the third dose of 4CMenB administered after completion of the last study visit procedures.

*4CMenB* four-component meningococcus capsular group B vaccine, *CI* confidence interval, *D* day, *hSBA* serum bactericidal assay using human complement, *M* number of participants with available data for the relevant endpoint, *MenACYW-TT* quadrivalent meningococcus ACYW tetanus toxoid-conjugate vaccine, *n* number of participants achieving hSBA seroprotection, *N* number of participants in the group in PPAS at D30 after vaccination at 12–13 months of age, *PPAS* per-protocol analysis set.

^a^Non-inferiority was demonstrated if the lower limits of the two-sided 95% CIs of the difference in seroprotection rates 30 days after the second vaccination at 12–13 months between Group 1 and Group 2 were > −10% against all four meningococcus capsular groups.

**Fig. 2:**
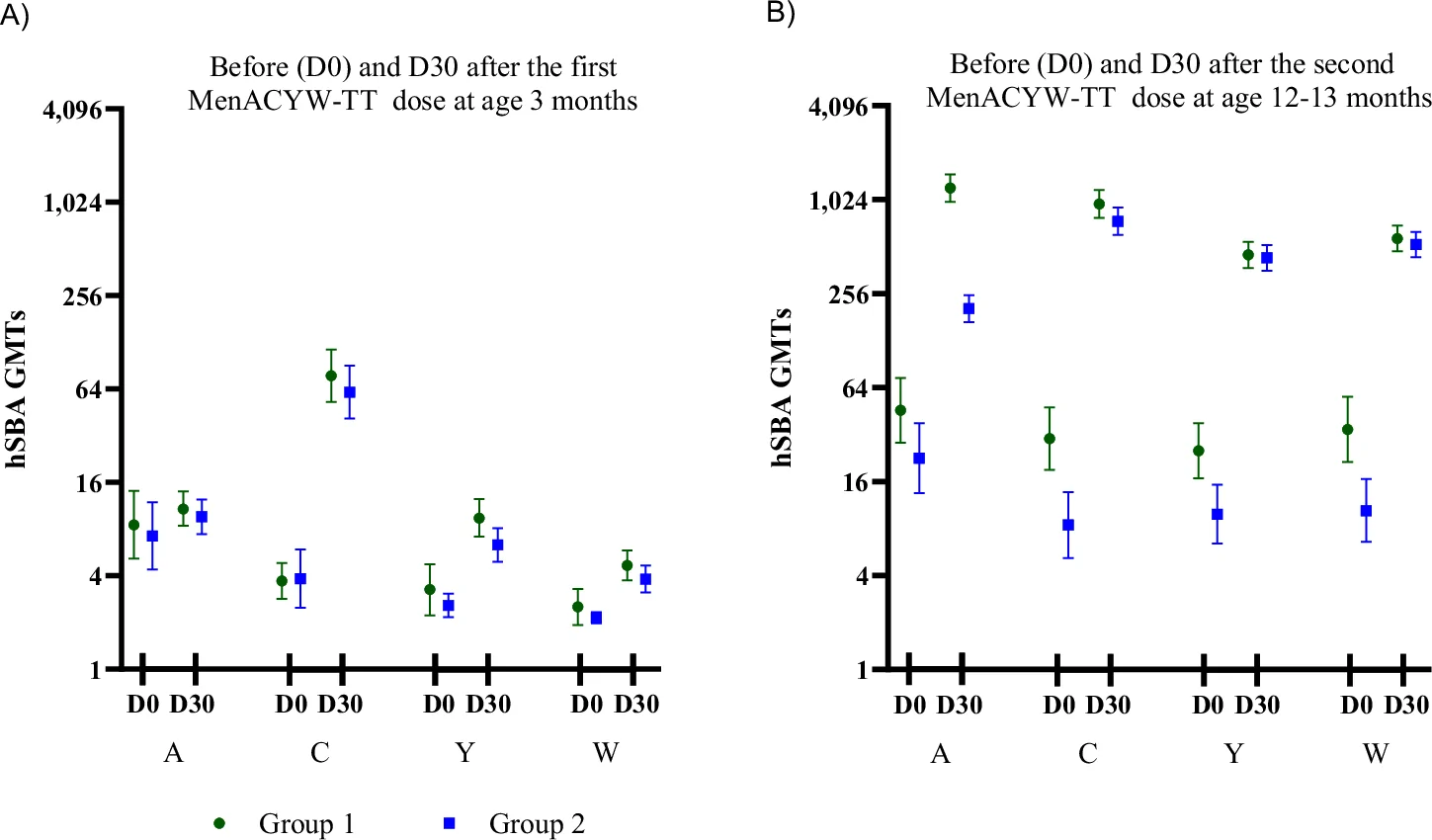
hSBA GMTs before (D0) and D30 after the vaccination at age 3 months and at age 12–13 months in Groups 1 and 2 (PPAS). Graphs show hSBA GMTs for meningococcus capsular groups A, C, Y, and W in Group 1 (green circle) and Group 2 (blue square), with bars representing 95% CIs: **A** before (D0) and 30 days after the first MenACYW-TT dose at 3 months of age and **B** before(D0) and 30 days after the second MenACYW-TT dose at 12–13 months of age. 4CMenB four-component meningococcus capsular group B vaccine, CI confidence interval, D day, GMT geometric mean titre, hSBA serum bactericidal assay using human complement, MenACYW-TT quadrivalent meningococcus ACYW tetanus toxoid-conjugate vaccine, PPAS per-protocol analysis set. Group 1: MenACYW-TT at 3 months and at 12–13 months of age; 4CMenB at 2, 4, and 12–13 months of age. Group 2: MenACYW-TT at 3 months and at 12–13 months of age; 4CMenB at 2 and 4 months of age, with the third dose of 4CMenB administered after completion of the last study visit procedures.

Capsular group-specific hSBA geometric mean titres (GMTs) before and after the first MenACYW-TT dose were very similar between Groups 1 and 2 (Fig. 2A). Interestingly, hSBA GMTs against capsular groups A, Y, and W were somewhat higher at age 12–13 months, before the second vaccine dose, than at 30 days after the first dose in both Groups 1 and 2. The hSBA GMT against capsular group A was higher in Group 1 than in Group 2 after the second MenACYW-TT dose (corresponding geometric mean titre ratio [GMTR] for Group 1/Group 2 was 5.9 [95% CI: 4.5; 7.8]) (Fig. 2B).

**Fig. 3:**
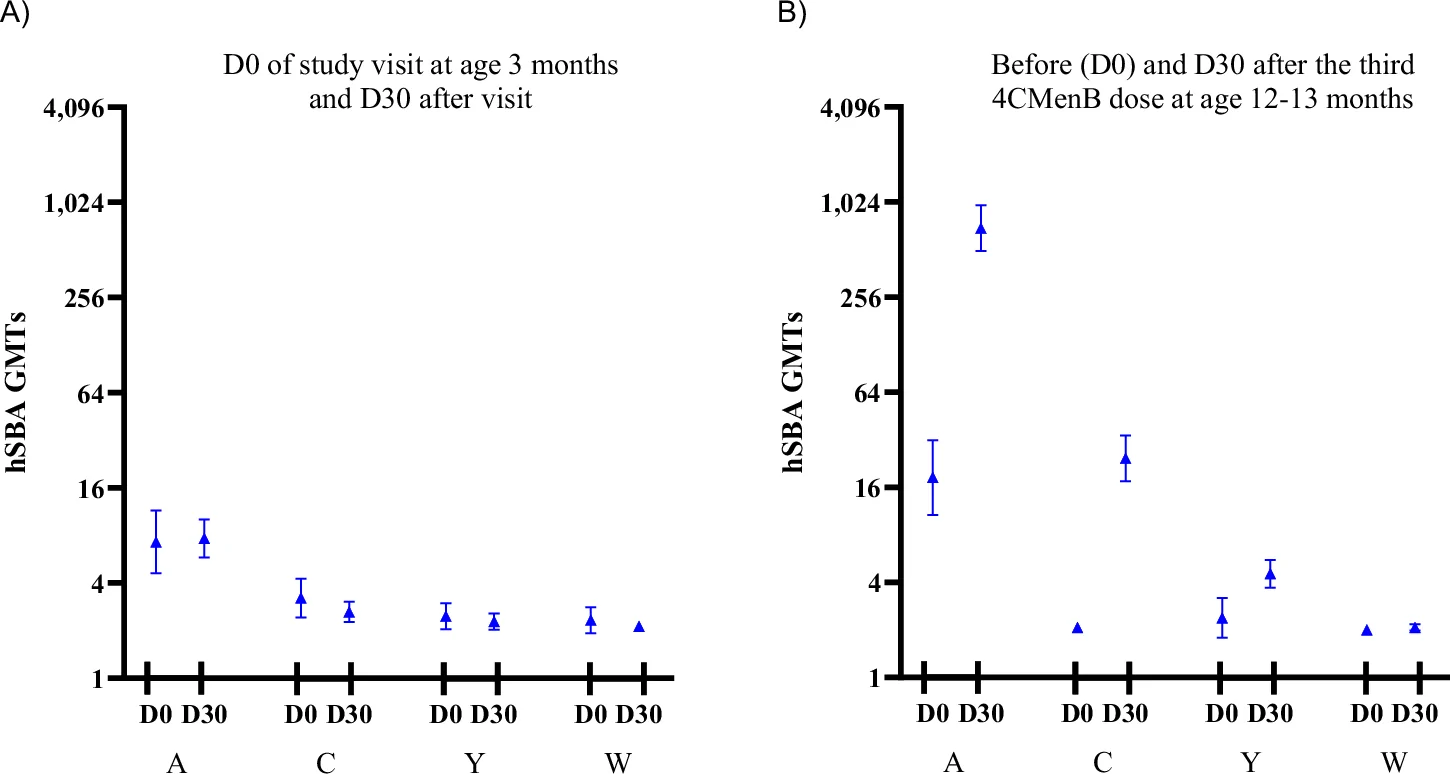
hSBA GMTs at D0 of the study visit and D30 after the vaccination at age 3 months and at age 12–13 months in Group 3 (PPAS). Graphs show hSBA GMTs for meningococcus capsular groups A, C, Y, and W in Group 3 (blue triangle), with bars representing 95% CIs: **A** on D0 and 30 days after the first vaccination visit at 3 months of age and **B** before (D0) and 30 days after the third 4CMenB dose at 12–13 months of age. 4CMenB four-component meningococcus capsular group B vaccine, CI confidence interval, D day, GMT geometric mean titre, hSBA serum bactericidal assay using human complement, PPAS per-protocol analysis set. Group 3: 4CMenB at 2, 4, and 12–13 months of age.

In Group 3, there were no significant changes in the hSBA GMTs against the four vaccine capsular groups between the first study vaccination visit at age 3 months (D0) and a month later (D30) (Fig. 3). There was then little change in these hSBA GMTs observed at age 12–13 months, apart from those against capsular group A, which rose. At 30 days after the 4CMenB booster, titres against capsular groups A, C, and Y rose noticeably, with a substantially higher increase against capsular group A.

**Fig. 4:**
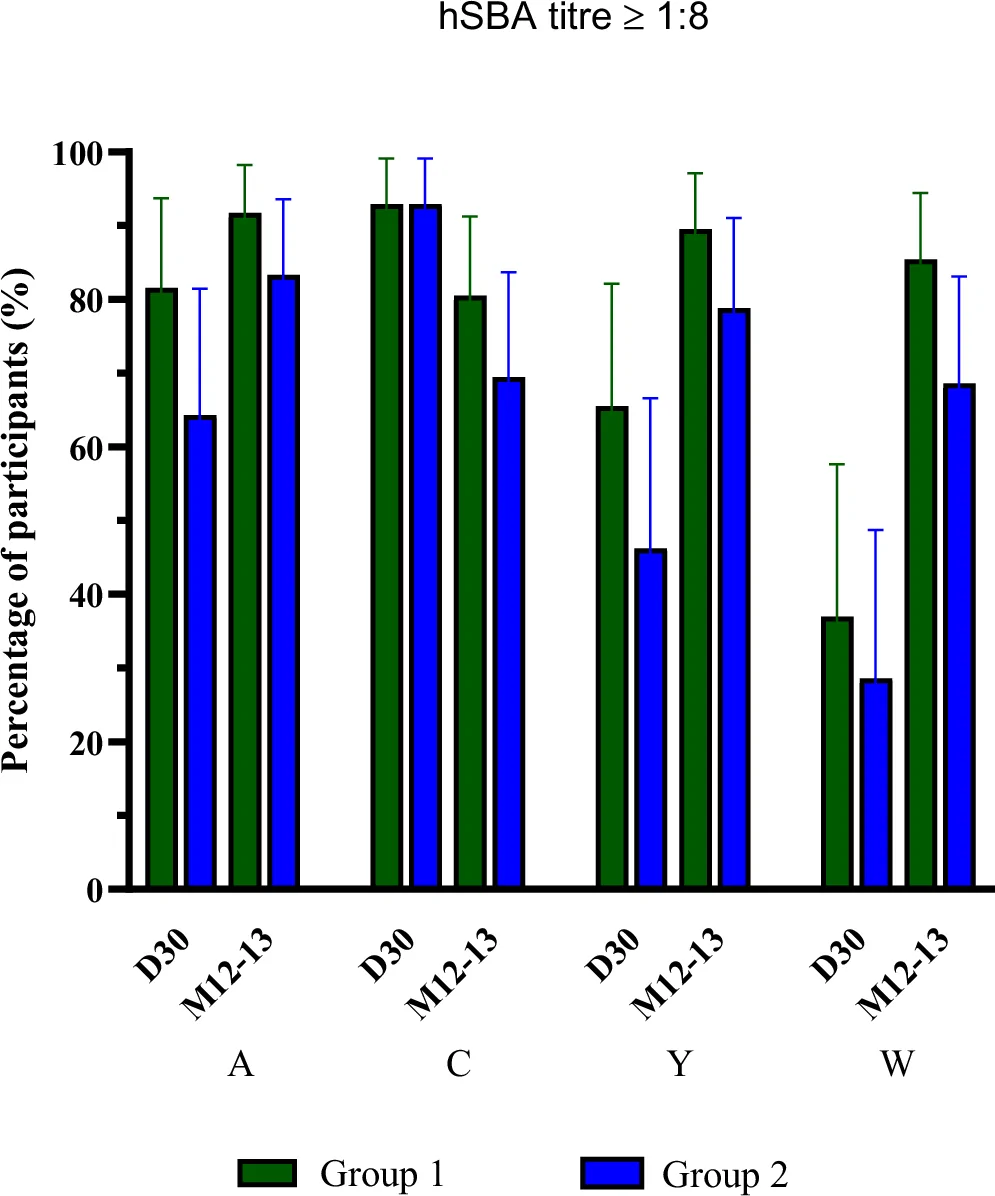
Persistence of hSBA titres ≥1:8 from D30 after the first vaccination at age 3 months to before the second vaccination at age 12–13 months in Groups 1 and 2 (PPAS). Graph shows proportions of participants with hSBA titres ≥1:8 for meningococcus capsular groups A, C, Y, and W at D30 after the first vaccination at age 3 months and before the second vaccination at age 12–13 months in Group 1 (green) and Group 2(blue), with 95% CIs. 4CMenB four-component meningococcus capsular group B vaccine, CI confidence interval, D day, hSBA serum bactericidal assay using human complement, M month, MenACYW-TT quadrivalent meningococcus ACYW tetanus toxoid-conjugate vaccine, PPAS per-protocol analysis set. Group 1: MenACYW-TT at 3 months and at 12–13 months of age; 4CMenB at 2, 4, and 12–13 months of age. Group 2: MenACYW-TT at 3 months and at 12–13 months of age; 4CMenB at 2 and 4 months of age, with the third dose of 4CMenB administered after completion of the last study visit procedures.

The proportion of participants with hSBA titres ≥1:4 and ≥1:8 in Groups 1 and 2 at baseline and at D30 after vaccination at 3 months of age is summarised in Supplementary Table S2 and after vaccination at 12–13 months in Supplementary Table S3. In concordance with the GMT findings, the proportion of participants with hSBA titres ≥1:4 and ≥1:8 appeared to rise between 30 days after the first dose and just before the second for capsular groups A, Y, and W (non-over lapping 95% CI for the latter only) but fell for group C (Fig. 4).

Serum bactericidal assay using rabbit complement (rSBA) GMTs against the four capsular groups did not differ significantly between Groups 1 and 2 at any of the timepoints assessed; of note, no enhanced responses against capsular group A were observed (Supplementary Fig. S1). However, in Group 3 (4CMenB only), there were increases in rSBA GMTs 30 days after the 12–13 month vaccination for capsular groups A and Y (Supplementary Table S4). These results should be interpreted with caution given the very low number of participants included in the assessments.

### Safety

**Fig. 5:**
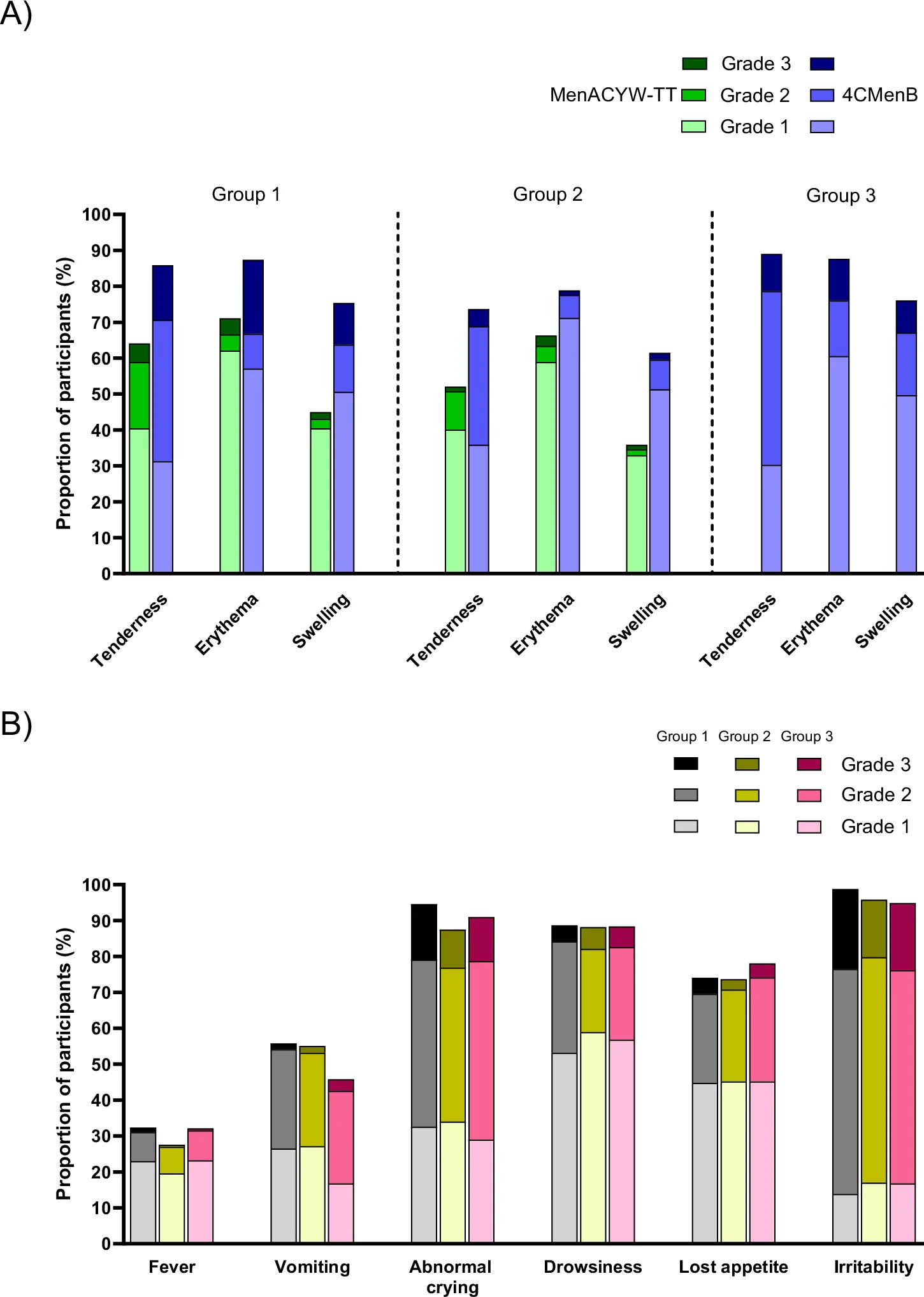
Solicited adverse reactions reported within 7 days after any vaccine injections, by maximum intensity during the solicited period (SafAS). **A** Injection site reactions in Groups 1, 2, and 3; green bars show injection site reactions following vaccination with MenACYW-TT, while blue bars show injection site reactions following vaccination with 4CMenB. **B** Systemic reactions in Groups 1, 2, and 3; reactions in Groups 1, 2, and 3 are presented as grey, yellow, and red bars, respectively. In both panels, bars represent the total proportion of participants experiencing each solicited reaction type, stratified by maximum severity; Grade 1 (mild), 2 (moderate) and 3 (severe) reactions are indicated by light, medium, and dark shading, respectively. 4CMenB four-component meningococcus capsular group B vaccine, MenACYW-TT quadrivalent meningococcus ACYW tetanus toxoid-conjugate vaccine, SafAS safety analysis set. Group 1: MenACYW-TT at 3 months and at 12–13 months of age; 4CMenB at 2, 4, and 12–13 months of age. Group 2: MenACYW-TT at 3 months and at 12–13 months of age; 4CMenB at 2 and 4 months of age, with the third dose of 4CMenB administered after completion of the last study visit procedures. Group 3: 4CMenB at 2, 4, and 12–13 months of age.

Immediate unsolicited adverse events (AEs) were uncommon, occurring in only one participant each in Group 2 (Grade 3 anaphylactic reaction) and Group 3 (adhesive tape allergy of unknown grade). The Grade 3 anaphylactic reaction occurred 10 min after MenACYW-TT administration at visit 4. The participant exhibited signs consistent with a severe allergic response, including cough, ocular erythema and oedema, wheezing, and generalised urticaria. The diagnosis of Grade 3 anaphylaxis was confirmed shortly after the second dose of the investigational vaccine. Immediate intervention involved intramuscular epinephrine administration and oral chlorphenamine maleate. No further doses of investigational vaccine were given. The proportion of participants who experienced AEs after any study vaccine is summarised in Table 3. Solicited injection site and systemic reactions were reported at similar rates across the three study groups (Fig. 5). Seventy serious adverse events (SAEs) were experienced by 59 participants: 5.7% [18/314], 10.8% [34/314], and 4.5% [7/157] of study vaccine recipients in Groups 1, 2, and 3, respectively. Of these, one participant (pyrexia) in Group 1 and three participants (petechiae, anaphylactic reaction, pyrexia) in Group 2 experienced SAEs that were considered related to study vaccines, but none led to study discontinuation. Among the adverse events of special interest (AESIs) monitored, there were five participants who reported seizures (described below). Based on the timing of the events and/or evidence of concomitant infections/underlying medical conditions, all AESIs were considered unrelated to study vaccines and resolved without any sequelae. There were no deaths reported during the study.

**Table 3:** Safety overview after any vaccine injection (SafAS)

Participants experiencing at least one:	Group 1 (*N* = 314)			Group 2 (*N* = 314)			Group 3 (*N* = 157)		
	*n*/*M*	%	(95% CI)	*n*/*M*	%	(95% CI)	*n*/*M*	%	(95% CI)
**Within 30** **min after any vaccine injections**:									
Immediate unsolicited AE	0/314	0	(0; 1.2)	1/314	0.3	(0; 1.8)	1/157	0.6	(0; 3.5)
Immediate unsolicited AR	0/314	0	(0; 1.2)	1/314	0.3	(0; 1.8)	0/157	0	(0; 2.3)
**Solicited reaction within 7 days after** **any vaccine injections**	310/310	100	(98.8; 100)	310/312	99.4	(97.7; 99.9)	155/155	100	(97.6; 100)
Solicited injection site reaction	305/310	98.4	(96.3; 99.5)	304/312	97.4	(95.0; 98.9)	153/155	98.7	(95.4; 99.8)
MenACYW-TT	270/309	87.4	(83.2; 90.9)	250/309	80.9	(76.1; 85.1)	-	-	-
4CMenB	296/310	95.5	(92.5; 97.5)	290/312	92.9	(89.5; 95.5)	151/155	97.4	(93.5; 99.3)
Solicited systemic reaction	308/310	99.4	(97.7; 99.9)	307/312	98.4	(96.3; 99.5)	151/155	97.4	(93.5; 99.3)
**Within 30 days after any vaccine injections:**									
Unsolicited AE	270/314	86.0	(81.6; 89.6)	258/314	82.2	(77.5; 86.2)	132/157	84.1	(77.4; 89.4)
Unsolicited AR	94/314	29.9	(24.9; 35.3)	84/314	26.8	(21.9; 32.0)	34/157	21.7	(15.5; 28.9)
AE leading to study discontinuation	2/314	0.6	(0.1; 2.3)	0/314	0	(0; 1.2)	0/157	0	(0; 2.3)
SAE	7/314	2.2	(0.9; 4.5)	20/314	6.4	(3.9; 9.7)	2/157	1.3	(0.2; 4.5)
Death	0/314	0	(0; 1.2)	0/314	0	(0; 1.2)	0/157	0	(0; 2.3)
AESI	1/314	0.3	(0; 1.8)	1/314	0.3	(0; 1.8)	0/157	0	(0; 2.3)
**During the study:**									
SAE	18/314	5.7	(3.4; 8.9)	34/314	10.8	(7.6; 14.8)	7/157	4.5	(1.8; 9.0)
Death	0/314	0	(0; 1.2)	0/314	0	(0; 1.2)	0/157	0	(0; 2.3)
AESI	2/314	0.6	(0.1; 2.3)	2/314	0.6	(0.1; 2.3)	1/157	0.6	(0; 3.5)

Group 1: MenACYW-TT at 3 months and at 12–13 months of age; 4CMenB at 2, 4, and 12–13 months of age.

Group 2: MenACYW-TT at 3 months and at 12–13 months of age; 4CMenB at 2 and 4 months of age, with the third dose of 4CMenB administered after completion of the last study visit procedures.

Group 3: 4CMenB at 2, 4, and 12–13 months of age.

*4CMenB* four-component meningococcus capsular group B vaccine, *AE* adverse event, *AESI* adverse event of special interest, *AR* adverse reaction, *MenACYW-TT* quadrivalent meningococcus ACYW tetanus toxoid-conjugate vaccine, *M* number of participants with available data for the endpoint, *N* number of participants in the overall SafAS for any dose; *n* number of participants in specified category, *SAE* serious adverse event, *SafAS* safety analysis set.

In Group 1, one participant had convulsions 185 days after 4CMenB vaccination associated with suspected gastrointestinal infection. A second participant in Group 1 had seizures 7 days after 4CMenB vaccination. Of note, this second participant had an underlying medical condition (diagnosis of multi-embolic ischaemic stroke, and electroencephalogram results which showed definite, moderate abnormality with an intermittent asymmetry between the two hemispheres). Thus, both the investigator and sponsor considered this event unrelated to vaccination. One participant in Group 2 with concomitant infection (tonsillitis) had a febrile seizure 13 days after MenACYW-TT vaccination. For this participant, the tonsillitis was considered the source of the fever associated with the febrile seizure. A second participant in Group 2 had a febrile seizure 193 days after 4CMenB vaccination associated with concomitant adenovirus infection. In Group 3, one participant had an afebrile seizure 95 days after 4CMenB vaccination.

### Sensitivity analysis

There were 145 participants (18.5%) affected by the COVID-19 pandemic. The main impact was on blood sampling, which could not be completed at Visits 3, 4, and 5. The impact of the COVID-19 pandemic on immunogenicity was considered negligible as <10% of the observations were affected. In addition, a comparable proportion of participants affected or not by the COVID-19 pandemic reported at least one solicited reaction (92.7% [127/137] vs 92.8% [568/612]) within 7 days after any vaccine injection in each treatment group. However, after the 12-to-13-month vaccination, fewer unsolicited AEs were reported in Groups 1, 2, and 3 by participants affected by the COVID-19 pandemic compared to those who were not affected (39.9% [57/143] vs 56.6% [349/617]). In Group 2, fewer participants affected by the COVID-19 pandemic compared to those not affected reported at least one unsolicited adverse reaction (AR) (1.7% [1/59] vs 10.9% [27/247]) or unsolicited non-serious AR (1.7% [1/59] vs 10.5% [26/247]).

## Discussion

Non-inferiority of MenACYW-TT administered concomitantly with 4CMenB (Group 1) compared with MenACYW-TT administered alone (Group 2) in the second year of life was demonstrated as regards hSBA seroprotection rates (proportion with antibody titre ≥1:8) against meningococcus capsular groups A, C, W, and Y. The proportion of participants who were seroprotected against capsular groups A, Y, and W increased between 30 days after the first MenACYW-TT dose given at age 3 months and age 12–13 months (before the second dose). This suggests that antibody titres against these meningococcal capsular antigens may have peaked later than the 30-day sample, perhaps enhanced by the 4CMenB dose given at 4 months of age. hSBA titres were substantially boosted after the second MenACYW-TT dose in both Groups 1 and 2.

The increase in hSBA GMTs against meningococcus capsular groups W and Y from one month post-dose 1 given at age 3 months to pre-dose 2 is consistent with that previously observed with another MenACWY-TT vaccine^12^. However, we also observed an increase in hSBA GMTs against capsular group A between one month post-dose 1 (given at age 3 months) and pre-dose 2 (at age 12–13 months). While a similar increase in capsular group A hSBA GMTs was not previously demonstrated with MenACWY-TT^12^, the infants in our study received a second dose of 4CMenB at 4 months of age, which may have contributed to cross-reactivity and increased responses to capsular group A. In fact, the *Neisseria* adhesin A (*nadA*) genes in capsular group A and C match that in 4CMenB^13^. However, the capsular group A strain used in both hSBA and rSBA assays demonstrates higher expression than capsular group C due to differences in the *nadA* gene promoter^13^.

It is well established that 4CMenB can induce bactericidal antibodies against non-B capsular group strains due to shared antigens across meningococcal capsular groups. Indeed, sera from 4CMenB-vaccinated individuals have exhibited bactericidal activity against meningococcus capsular groups A, C, W, Y, and X clinical isolates^14–17^. Consistent with these findings, we observed increased bactericidal activity against meningococcus capsular groups A, C, and Y (in descending order), but not W, following three doses of 4CMenB in Group 3. Nonetheless, the data clearly demonstrate orders-of-magnitude differences in responses against meningococcus capsular groups C, W, and Y when MenACYW-TT was administered (Groups 1 and 2) versus 4CMenB alone (Group 3), highlighting that MenACYW-TT vaccination is essential for robust immune responses against these capsular types and represents the best approach for capsular group-specific protection.

A high proportion of participants in this study exhibited pre-existing meningococcus capsular group A hSBA titres prior to MenACWY-TT vaccination (Fig. 2A). Pre-dose 1 GMTs were 8.52 and 7.22 in Groups 1 and 2, respectively, and 7.27 in Group 3 (4CMenB alone) at 3 months of age. These values were substantially elevated compared with the baseline meningococcus capsular group A hSBA GMT of 3.0 observed in unimmunised infants assessed in the same laboratory^18^, suggesting that prior 4CMenB vaccination may have contributed to the elevated pre-vaccination titres observed across all groups.

Interestingly, although we observed enhanced cross-reactivity between capsular group A and 4CMenB after a second dose of MenACYW-TT with concomitant 4CMenB, which was greater in Group 1 than Group 2, likely reflecting the additive immunogenic contribution of co-administering both vaccines, this enhanced response was not detected with rSBA when comparing Group 1 to Group 2. However, participants who received 4CMenB only (Group 3) demonstrated increases in rSBA GMTs for capsular groups A and Y 30 days after the 12–13-month vaccination. While the rSBA results must be interpreted with caution due to the low sample size, these data support cross-reactivity of 4CMenB against non-B capsular group strains.

MenACYW-TT administered concomitantly with 4CMenB (Group 1) had a similar safety profile to that of MenACYW-TT administered alone. However, MenACYW-TT (at age 3 months and 12–13 months, i.e. two doses) appears slightly more reactogenic in the current study than previously reported in older infants (12–23 months, single dose)^19–21^. This is perhaps expected since the participants in the current study received two study vaccines, whereas in the other studies, only one vaccine dose was administered. Nonetheless, no new safety concerns were identified with any of the study vaccines. A single case of anaphylaxis, assessed as related to MenACYW-TT, was reported. Anaphylaxis is a known possibility with any vaccination and was not considered a new safety concern. This represents the only case of anaphylaxis assessed as related to MenACYW-TT among over 6000 participants aged 6 weeks to 18 months who received at least one dose across the clinical development program.

Due to the occurrence of the COVID-19 pandemic during the study and the impact of the pandemic on 18.5% of participants, a sensitivity analysis was performed to assess the impact on study conduct. We identified a decrease in the number of blood samples drawn during the COVID-19 pandemic, probably associated with reallocation of staff to COVID-19 efforts and potentially COVID-19 restrictions impacting participant visits. There were also some differences in the reporting of AEs between those impacted by the COVID-19 pandemic versus those who were not. However, no safety concerns were identified. Overall, the impact of the COVID-19 pandemic on study conduct was well managed through mitigation efforts by the sites and operational teams.

Our study has several limitations to consider. The study was open-label since there were differences in vaccine regimens between the two study groups, which would have made using a double-blind or even an observer-blind study design challenging. The rSBA data before the first and the second doses need to be interpreted with caution due to the limited sample size at these timepoints. Immune responses to other paediatric vaccines administered per the routine schedule were not assessed in this study. However, existing evidence demonstrates that MenACWY-TT co-administration does not compromise the safety or immunogenicity of routine paediatric vaccines in infants and toddlers^9,18,19^. Although potential interference of MenACWY-TT with 4CMenB antibody responses was not formally evaluated, the primary 4CMenB series (2 and 4 months of age) was administered without concomitant MenACWY-TT, minimising concerns regarding the primary vaccination schedule. Nonetheless, given that MenACWY-TT and the 4CMenB booster are both administered at 12–13 months of age, future studies evaluating potential immunological interactions at this time point are warranted. Study strengths include that the hSBA and rSBA were each conducted at single laboratories, respectively, both of which have extensive experience with these assays.

In conclusion, MenACYW-TT exhibited comparable immunogenicity and safety when co-administered with 4CMenB or given alone, supporting its use within a 1 + 1 schedule at 3 and 12–13 months of age in line with the UK National Immunisation Programme. Given the ongoing global expansion of 4CMenB vaccination, these findings provide timely evidence to guide the integration of MenACYW-TT into diverse national immunisation strategies.

## Methods

### Study design and participants

We performed a phase III, open-label, randomised, parallel-group, active-controlled, multi-centre study (NCT03632720) at 16 sites in the UK from 10 October 2018 to 5 December 2022. We recruited infants ≥2 months of age (age range of 56 days to 89 days) on the day of the first study visit. These infants had to have been born at full term (≥37 weeks gestation) with a birth weight ≥2.5 kg and not have been previously vaccinated against meningococcal disease with either mono- or polyvalent, polysaccharide, or conjugate meningococcal vaccines containing any of capsular groups A, B, C, W, and Y or with any pneumococcal, diphtheria, tetanus, pertussis, hepatitis B, *Haemophilus influenzae* type b (Hib), poliovirus or rotavirus vaccines.

The study was undertaken in compliance with the International Conference on Harmonisation guidelines for Good Clinical Practice and the principles of the Declaration of Helsinki. Informed consent was obtained from participants’ parents (or legal guardians) before any study procedures were performed. The protocol and all amendments were approved by Yorkshire and the Humber–Sheffield Research Ethics Committee (reference number: 18/YH/0223) and appropriate regulatory agency.

### Randomisation

Participants were centrally randomised in blocks of 5 without stratification to one of three groups via an interactive response technology system in a 2:2:1 ratio to receive MenACYW-TT (MenQuadfi®; Sanofi, Swiftwater, PA, USA) at 3 and at 12–13 months of age and the four-component meningococcus capsular group B vaccine (4CMenB; Bexsero®; GlaxoSmithKline, Rixensart, Belgium) at 2, 4, and 12–13 months of age (Group 1), MenACYW-TT at 3 and at 12–13 months of age and 4CMenB at 2 and 4 months of age (Group 2), or 4CMenB only at 2, 4, and 12–13 months of age (Group 3).

### Procedures

MenACYW-TT was provided as a fully liquid formulation in 0.5 mL single-dose vials and administered intramuscularly into the anterolateral area of the thigh. Each 0.5 mL of MenACYW-TT contained 10 μg doses of each of meningococcus capsular groups A, C, W, and Y polysaccharides conjugated to approximately 55 μg tetanus toxoid carrier. 4CMenB was provided as supplied as a suspension for injection in pre-filled syringes (0.5 mL). Each 0.5 mL of 4CMenB contained 50 μg of each of the following recombinant *Neisseria meningitidis* capsular group B proteins: NHBA fusion, NadA and fHbp fusion, and 25 μg of outer membrane vesicles from *N meningitidis* capsular group B strain NZ98/254. 4CMenB was administered by deep intramuscular injection in the anterolateral aspect of the thigh in infants.

All participants were kept under observation to monitor for any immediate AEs occurring within 30 minutes after any study vaccine. Parents/legal guardians were provided with a diary card, a digital thermometer, and a flexible ruler and were instructed to record daily body temperature and solicited injection site (tenderness, erythema, and swelling) and systemic reactions (fever, vomiting, abnormal crying, drowsiness, loss of appetite, and irritability) for 7 days, as well as unsolicited AEs for 30 days after each study injection. Parents/legal guardians also graded all AEs and ARs on a three-point scale (except measurable reactions of erythema, swelling, and fever, which were graded during analysis) as described elsewhere^22^. SAEs were recorded throughout the study. Investigators assigned the causal relationship (related or not related) to study vaccine for each unsolicited AE and SAE. AESIs solicited were generalised seizures (febrile and non-febrile), Kawasaki disease, Guillain-Barré syndrome, and idiopathic thrombocytopenic purpura.

The schedule of study blood sample collection is summarised in Supplementary Table S1. Blood samples (approximately 3 mL) for immunogenicity assessment were collected in a staggered manner from a subset of participants in Groups 1 and 2 (about 70 participants each) before the first vaccination (at 3 months) and 30 days after the first (4 months) and second (13–14 months) vaccinations and from another subset of participants in Groups 1 and 2 (about 90 participants each) at 30 days after the first (4 months) and before (12–13 months) and 30 days after the second (13–14 months) vaccinations. The remainder of participants in Groups 1 and 2 only provided one blood sample 30 days after the second vaccination (13–14 months). Blood samples were collected in a similar staggered manner for participants in Group 3: a subset (about 70 participants planned [*n* = 51 actual]) before the first vaccination (at 3 months) and 30 days after the first (4 months) and second (13–14 months) vaccinations and another subset (about 90 participants each) at 30 days after the first (4 months) and before (12–13 months) and after the second (13–14 months) vaccinations. These blood samples were allowed to clot at room temperature, and the serum was separated and stored at –20 ^o^C until laboratory analysis.

Routine paediatric vaccines were administered to participants in all groups according to the UK immunisation schedule (Supplementary Table S1). Infants in Group 2 received their third dose of 4 CMenB after completion of the last study visit procedures. No additional immunogenicity samples and/or safety data were collected after the administration of routine vaccines, beyond those described above.

The protocol neither mandated nor prohibited the use of analgesics, including paracetamol, allowing sites to follow standard UK clinical practice. In the UK National Immunisation Programme, prophylactic paracetamol is routinely recommended with the first two doses of 4CMenB (administered at 2 and 4 months of age) to reduce the risk of fever, but it is not routinely recommended with the toddler booster dose at 12-13 months. All paracetamol use was recorded as a concomitant medication and monitored throughout the study.

### Immunogenicity assessment methods

Functional meningococcal antibody activity against capsular groups A, C, W, and Y was measured with a validated hSBA (reference strains: A [F8238], C [C-11], Y [3021], and W-135 [2515]) at a single centre, Global Clinical Immunology (Sanofi, Swiftwater, PA, USA), as described in detail elsewhere^23^. An rSBA (reference strains: A [8238], C [C-11], Y [M03 241125], and W [M01 240070]) was also undertaken in a similar manner, at the UK Health Security Agency Vaccine Evaluation Unit (Manchester, UK) on a subset consisting of the first 100 participants randomised to have three blood samples in each of the 3 study groups.

### Outcomes

The primary endpoint was antibody titres ≥1:8 against meningococcus capsular groups A, C, W, and Y measured by hSBA assessed 30 days after vaccination at 12–13 months of age (Group 1 versus Group 2). Secondary immunogenicity endpoints included hSBA GMTs against capsular groups A, C, W, and Y before and 30 days after vaccinations with MenACYW-TT concomitantly with 4CMenB or alone, and rSBA GMTs in a subset of Group 1, proportion with hSBA titres ≥1:4 and ≥1:8 and proportion with rSBA titres ≥1:8 and ≥1:128. Seroresponse was defined as post-vaccination titre ≥1:16 (or ≥1:32 for rSBA) for participants with pre-vaccination titres <1:8, and a ≥4-fold post-vaccination titre rise for participants with pre-vaccination titres ≥1:8.

Safety was an observational endpoint and included the occurrence, nature, and severity of injection site and systemic solicited ARs up to 7 days after each vaccination, and unsolicited AEs up to 30 days after each vaccination, and SAEs (including AESIs) through the end of the trial.

### Statistical analyses

The primary objective was to demonstrate the non-inferiority of the antibody responses to meningococcus capsular groups A, C, W, and Y in terms of seroprotection rates (proportion with hSBA titres ≥1:8 against each of the four capsular groups) when MenACYW-TT was administered concomitantly with 4CMenB (Group 1) in the second year of life compared to when MenACYW-TT was given alone (Group 2). Non-inferiority was demonstrated if the lower limit of the two-sided 95% CI, calculated using the Wilson Score method without continuity correction^24^, for the difference in seroprotection rates 30 days after the second vaccination at 12–13 months between Group 1 and Group 2 was > −10% against all four meningococcus capsular groups.

An initial sample size of approximately 700 participants was considered, but due to the potential impact of the COVID-19 pandemic, an additional 100 participants were enrolled to ensure sufficient study power for the primary objective. Thus, approximately 800 participants were enrolled and assuming a 30% non-adherence rate, would result in about 560 participants (224 in Group 1, 224 in Group 2, and 112 in Group 3) in the per-protocol population available for immunogenicity analyses. With 224 evaluable participants each in Group 1 and Group 2, the study would have 88% overall power to declare the non-inferiority of Group 1 versus Group 2 based on seroprotection rates (proportion with hSBA titre ≥1:8) against capsular groups A, C, W, and Y.

hSBA and rSBA antibodies were summarised as GMTs and GMTRs (between Group 1 and Group 2) with corresponding 95% CIs for each of the four capsular groups. These were calculated assuming that the log10 transformation of the titres followed a normal distribution, then anti-log transformation applied to the results of mean and 95% CI calculated from normal distribution to provide GMTs and associated 95% CIs on their original scale. The 95% CI for the hSBA GMTR between Groups 1 and 2 was calculated by taking the antilogarithms of the lower and upper limits of the 95% CI for the difference in log(GMT) between the two groups. Unsolicited AEs and SAEs were summarised by System Organ Class and Preferred Term using the Medical Dictionary for Regulatory Activities. Categorical variables were summarised as frequency counts, percentages, and 95% CIs, calculated using the exact binomial distribution (Clopper-Pearson method)^25^.

The populations analysed included the PPAS and the SafAS, with no imputation for missing data and outliers. The immunogenicity analyses were performed on the PPAS. The PPAS included participants with no relevant protocol deviations and a valid post-vaccination serology result. The PPAS was also divided into three sets depending on whether the participants had a valid post-vaccination serology (after the first vaccination, and before and after the second vaccination). The SafAS included all participants who received at least one dose of the study vaccine and had associated safety data available.

A sensitivity analysis was planned if >10% of the participants were impacted by the COVID-19 pandemic, which included those with at least one major or critical protocol deviation or those unable to complete the study due to pandemic-related restrictions. If the 10% threshold was reached, then the main immunogenicity and safety endpoints were to be summarised for these affected and non-affected participants.

Statistical analyses were done using SAS® Version 9.4 software or later (SAS Institute Inc, Cary, NC, USA).

## Supplementary information

Supplementary Material

## Data Availability

The datasets generated and/or analysed during the current study are not publicly available due to regulatory and ethical obligations, including the protection of participant privacy, as well as confidential information and responsible data use considerations, but are available from the corresponding author on reasonable request. Qualified researchers may request access to patient-level data and related documents, including, for example, the clinical study report, study protocol with any amendments, blank case report form, statistical analysis plan, and dataset specifications. Patient-level data will be anonymised, and study documents will be redacted to protect the privacy of trial participants. Further details on Sanofi’s data sharing criteria, eligible studies, and process for requesting access can be found at https://vivli.org/.
