# Supplementary material for: Safety and immunogenicity of concomitant quadrivalent meningococcus ACYW-TT and meningococcus group B vaccines
Source: NPJ Vaccines. 2026 Jul 1;11:195. doi: 10.1038/s41541-026-01508-7 (PMC13687022; doi:10.1038/s41541-026-01508-7)
Supplement: Supplementary file 1 [file 41541_2026_1508_MOESM1_ESM.pdf]

Supplemental Material

Table S1. Schedule of vaccination and blood sample collection

Group	Visit								
	**Visit 1 2 months**	**Visit 2 3 months**		**Visit 3 4 months**		**Visit 4 12–13 months**		**Visit 5 13–14 months**	
	**Vaccinations**	**Blood collection**	**Vaccinations**	**Blood collection**	**Vaccinations**	**Blood collection**	**Vaccination(s)**	**Blood collection**	**Vaccinations***
**1 (N=320)**	Infanrix hexa Rotarix Bexsero Prevenar 13	**BL1** (n=70)†	Infanrix hexa Rotarix **MenACYW-TT**	**BL2** (n=70)†	Infanrix hexa Bexsero Prevenar 13		Bexsero **MenACYW-TT**	**BL3** (n=320)	Prevenar 13 Menitorix Priorix/M-M-RVAXPRO
				**BL1** (n=90)‡		**BL2** (n=90)‡			
**2 (N=320)**	Infanrix hexa Rotarix Bexsero Prevenar 13	**BL1** (n=70)†	Infanrix hexa Rotarix **MenACYW-TT**	**BL2** (n=70)†	Infanrix hexa Bexsero Prevenar 13		**MenACYW-TT**	**BL3** (n=320)	Prevenar 13 Menitorix Bexsero§ Priorix/M-M-RVAXPRO
				**BL1** (n=90)‡		**BL2** (n=90)‡			
**3** ** **(N=160)**	Infanrix hexa Rotarix Bexsero Prevenar 13	**BL1** (n=70)†	Infanrix hexa Rotarix	**BL2** (n=70)†	Infanrix hexa Bexsero Prevenar 13		Bexsero	**BL3** (n=160)	Prevenar 13 Menitorix Priorix/M-M-RVAXPRO
				**BL1** (n=90)‡		**BL2** (n=90)‡			

*Routine vaccines were given after study visit procedures were completed. No additional immunogenicity or safety data were collected after the administration of these vaccines.

†The first ~70 participants randomised to have 2 blood draws at Visit 2 and Visit 3 (Subset A).

[‡]The last ~90 participants randomised to have 2 blood draws at Visit 3 and Visit 4 (Subset B).

[§]For Group 2, the 3rd dose of Bexsero was provided for completion of the Bexsero vaccination series.

[**]Licensed ACWY conjugate meningococcus vaccine (Nimenrix®) was offered at least 30 days after the last study visit (Visit 5) at an additional optional visit.

4CMenB, four-component meningococcus capsular group B vaccine; BL, blood draw prior to vaccination; MenACYW-TT, quadrivalent meningococcus ACYW tetanus toxoid-conjugate vaccine; N, number of participants randomised in each group; n, number of participants randomised to have blood draws in each group at the given visit.

Group 1: MenACYW-TT at 3 months and at 12–13 months of age; 4CMenB at 2, 4, and 12–13 months of age.

Group 2: MenACYW-TT at 3 months and at 12–13 months of age; 4CMenB at 2 and 4 months of age, with the third dose of 4CMenB administered after completion of the last study visit procedures.

Group 3: 4CMenB at 2, 4, and 12–13 months of age.

Table S2. Summary of hSBA titres ≥1:4 and ≥1:8 at baseline and at D30 after vaccination at 3 months of age (PPAS)

			Group 1 (N=78)		Group 2 (N=77)	
**Subgroup**	**Time point**	**hSBA titres**	**n/M**	**% (95% CI)**	**n/M**	**% (95% CI)**
A	D0	≥1:4	18/22	81.8 (59.7; 94.8)	20/27	74.1 (53.7; 88.9)
		≥1:8	13/22	59.1 (36.4; 79.3)	13/27	48.1 (28.7; 68.1)
	D30	≥1:4	56/64	87.5 (76.8; 94.4)	61/71	85.9 (75.6; 93.0)
		≥1:8	47/64	73.4 (60.9; 83.7)	47/71	66.2 (54.0; 77.0)
C	D0	≥1:4	15/27	55.6 (35.3; 74.5)	13/34	38.2 (22.2; 56.4)
		≥1:8	7/27	25.9 (11.1; 46.3)	7/34	20.6 (8.7; 37.9)
	D30	≥1:4	72/77	93.5 (85.5; 97.9)	70/77	90.9 (82.2; 96.3)
		≥1:8	67/77	87.0 (77.4; 93.6)	68/77	88.3 (79.0; 94.5)
Y	D0	≥1:4	8/27	29.6 (13.8; 50.2)	8/30	26.7 (12.3; 45.9)
		≥1:8	6/27	22.2 (8.6; 42.3)	3/30	10.0 (2.1; 26.5)
	D30	≥1:4	58/71	81.7 (70.7; 89.9)	47/68	69.1 (56.7; 79.8)
		≥1:8	47/71	66.2 (54.0; 77.0)	37/68	54.4 (41.9; 66.5)
W	D0	≥1:4	4/24	16.7 (4.7; 37.4)	3/29	10.3 (2.2; 27.4)
		≥1:8	2/24	8.3 (1.0; 27.0)	0/29	0 (0; 11.9)

D30	≥1:4	43/76	56.6 (44.7; 67.9)	34/73	46.6 (34.8; 58.6)
	≥1:8	27/76	35.5 (24.9; 47.3)	20/73	27.4 (17.6; 39.1)

CI, confidence interval; D, day; hSBA, serum bactericidal assay using human complement; M, number of participants with available data for relevant endpoint; N, number of participants in PPAS for immunogenicity evaluation after infant vaccination; n, number of participants experiencing the endpoint listed; PPAS, per protocol analysis set.

Group 1: MenACYW-TT at 3 months and at 12–13 months of age; 4CMenB at 2, 4, and 12–13 months of age.

Group 2: MenACYW-TT at 3 months and at 12–13 months of age; 4CMenB at 2 and 4 months of age, with the third dose of 4CMenB

Table S3. Summary of hSBA titres ≥1:4 and ≥1:8 at baseline and at D30 after vaccination at 12–13 months of age (PPAS at D30 after vaccination at 12–13 months of age)

			Group 1 (N=178)		Group 2 (N=169)	
**Subgroup**	**Time point**	**hSBA titres**	**n/M**	**% (95% CI)**	**n/M**	**% (95% CI)**
A	D0	≥1:4	31/31	100 (88.8; 100)	25/26	96.2 (80.4; 99.9)
		≥1:8	29/31	93.5 (78.6; 99.2)	20/26	76.9 (56.4; 91.0)
	D30	≥1:4	159/159	100 (97.7; 100)	156/156	100 (97.7; 100)
		≥1:8	159/159	100 (97.7; 100)	155/156	99.4 (96.5; 100)
C	D0	≥1:4	33/35	94.3 (80.8; 99.3)	19/26	73.1 (52.2; 88.4)
		≥1:8	30/35	85.7 (69.7; 95.2)	14/26	53.8 (33.4; 73.4)
	D30	≥1:4	168/168	100 (97.8; 100)	165/165	100 (97.8; 100)
		≥1:8	168/168	100 (97.8; 100)	165/165	100 (97.8; 100)
Y	D0	≥1:4	31/32	96.9 (83.8; 99.9)	23/26	88.5 (69.8; 97.6)
		≥1:8	30/32	93.8 (79.2; 99.2)	19/26	73.1 (52.2; 88.4)
	D30	≥1:4	161/161	100 (97.7; 100)	159/159	100 (97.7; 100)
		≥1:8	161/161	100 (97.7; 100)	159/159	100 (97.7; 100)
W	D0	≥1:4	34/35	97.1 (85.1; 99.9)	22/26	84.6 (65.1; 95.6)

		≥1:8	31/35	88.6 (73.3; 96.8)	18/26	69.2 (48.2; 85.7)
	D30	≥1:4	171/172	99.4 (96.8; 100)	160/160	100 (97.7; 100)
		≥1:8	171/172	99.4 (96.8; 100)	160/160	100 (97.7; 100)

CI, confidence interval; D, day; hSBA, serum bactericidal assay using human complement; M, number of participants with available data for relevant endpoint; N, number of participants in PPAS at D30 after vaccination at 12–13 months of age; n, number of participants experiencing the endpoint listed; PPAS, per protocol analysis set.

Group 1: MenACYW-TT at 3 months and at 12–13 months of age; 4CMenB at 2, 4, and 12–13 months of age.

Group 2: MenACYW-TT at 3 months and at 12–13 months of age; 4CMenB at 2 and 4 months of age, with the third dose of 4CMenB

Table S4. Summary of geometric means of rSBA titres for Group 3 – rSBA subset (PPAS)

**Subgroup**	**Time point**	**M**	**GMT**	**(95% CI)**
A	D0 (before 3-month vaccination)	25	2.00	(NC; NC)
	D30 (after 3-month vaccination)	36	2.04	(1.96; 2.12)
	D0 (before 12–13-month vaccination)	6	32.0	(0.99; 1032)
	D30 (after 12–13-month vaccination)	41	391	(181; 845)
C	D0 (before 3-month vaccination)	26	2.54	(1.98; 3.26)
	D30 (after 3-month vaccination)	37	2.50	(2.00; 3.14)
	D0 (before 12–13-month vaccination)	6	2.00	(NC; NC)
	D30 (after 12–13-month vaccination)	44	3.16	2.13; 4.68)
Y	D0 (before 3-month vaccination)	26	2.75	(1.96; 3.86)
	D30 (after 3-month vaccination)	31	2.92	(1.78; 4.82)
	D0 (before 12–13-month vaccination)	6	2.00	(NC; NC)
	D30 (after 12–13-month vaccination)	43	742	(418; 1315)
W	D0 (before 3-month vaccination)	26	2.29	(1.74; 3.01)
	D30 (after 3-month vaccination)	36	2.00	(NC; NC)
	D0 (before 12–13-month vaccination)	6	2.00	(NC; NC)
	D30 (after 12–13-month vaccination)	42	4.13	(2.49; 6.86)

CI, confidence interval; D, day; GMT, geometric mean titre; M, number of participants with available data for the endpoint; NC, not computed; rSBA, serum bactericidal assay using rabbit complement; PPAS, per-protocol analysis set.

Figure S1. rSBA GMTs at before (D0) and D30 after vaccination at 3 months (A) and at 12–13 months of age (B) in Group 1 and Group 2[#] (rSBA subset PPAS)

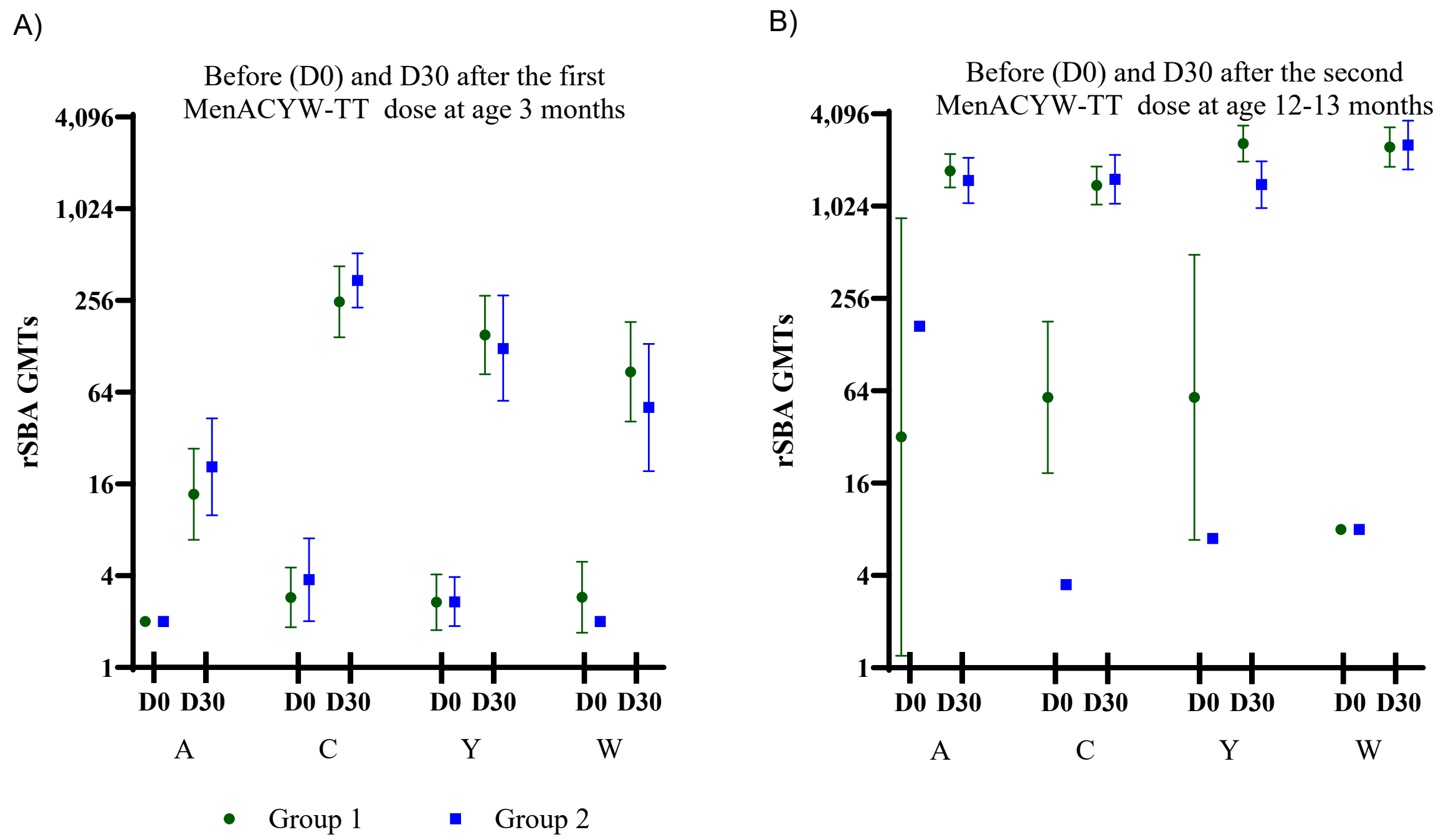

[#]D0 data should be interpreted with caution due to the small sample size. When 95% CIs could not be calculated, they are not displayed.

4CMenB, four-component meningococcus capsular group B vaccine; CI, confidence interval; D, day; GMT, geometric mean titre; MenACYW-TT, quadrivalent meningococcus ACYW tetanus toxoid-conjugate vaccine; PPAS, per-protocol analysis set; rSBA, serum bactericidal assay using rabbit complement.

Group 1: MenACYW-TT at 3 months and at 12–13 months of age; 4CMenB at 2, 4, and 12–13 months of age.

Group 2: MenACYW-TT at 3 months and at 12–13 months of age; 4CMenB at 2 and 4 months of age, with the third dose of 4CMenB administered after completion of the last study visit procedures.

CONSORT 2025 checklist of information to include when reporting a randomised trial*

Section / Topic	No	CONSORT 2025 checklist item description	Reported on page no.
**Title and abstract**			
Title and structured abstract	1a	Identification as a randomised trial	3
	1b	Structured summary of the trial design, methods, results, and conclusions	3
**Open science**			
Trial registration	2	Name of trial registry, identifying number (with URL) and date of registration	3
Protocol and statistical analysis plan	3	Where the trial protocol and statistical analysis plan can be accessed	24
Data sharing	4	Where and how the individual de-identified participant data (including data dictionary), statistical code and any other materials can be accessed	24
Funding and conflicts of interest	5a	Sources of funding and other support (e.g., supply of drugs), and role of funders in the design, conduct, analysis and reporting of the trial	23
	5b	Financial and other conflicts of interest of the manuscript authors	23-24
**Introduction**			
Background and rationale	6	Scientific background and rationale	4-5
Objectives	7	Specific objectives related to benefits and harms	5
**Methods**			
Patient and public involvement	8	Details of patient or public involvement in the design, conduct and reporting of the trial	NA
Trial design	9	Description of trial design including type of trial (e.g., parallel group, crossover), allocation ratio, and framework (e.g., superiority, equivalence, non-inferiority, exploratory)	16-19
Changes to trial protocol	10	Important changes to the trial after it commenced including any outcomes or analyses that were not prespecified, with reason	NA
Trial setting	11	Settings (e.g., community, hospital) and locations (e.g., countries, sites) where the trial was conducted	16
Eligibility criteria	12a	Eligibility criteria for participants	16
	12b	If applicable, eligibility criteria for sites and for individuals delivering the interventions (e.g., surgeons, physiotherapists)	NA

Intervention and comparator	13	Intervention and comparator with sufficient details to allow replication. If relevant, where additional materials describing the intervention and comparator (e.g., intervention manual) can be accessed	17-18
Outcomes	14	Pre-specified primary and secondary outcomes, including the specific measurement variable (e.g., systolic blood pressure), analysis metric (e.g., change from baseline, final value, time to event), method of aggregation (e.g., median, proportion), and time point for each outcome	19-20
Harms	15	How harms were defined and assessed (e.g., systematically, non-systematically)	17-18
Sample size	16a	How sample size was determined, including all assumptions supporting the sample size calculation	20-21
	16b	Explanation of any interim analyses and stopping guidelines	NA
Randomisation:			
Sequence generation	17a	Who generated the random allocation sequence and the method used	16-17
	17b	Type of randomisation and details of any restriction (e.g., stratification, blocking and block size)	16-17
Allocation concealment mechanism	18	Mechanism used to implement the random allocation sequence (e.g., central computer/telephone; sequentially numbered, opaque, sealed containers), describing any steps to conceal the sequence until interventions were assigned	16-17
Implementation	19	Whether the personnel who enrolled and those who assigned participants to the interventions had access to the random allocation sequence	
Blinding	20a	Who was blinded after assignment to interventions (e.g., participants, care providers, outcome assessors, data analysts)	NA (open-label study)
	20b	If blinded, how blinding was achieved and description of the similarity of interventions	
Statistical methods	21a	Statistical methods used to compare groups for primary and secondary outcomes, including harms	20-22
	21b	Definition of who is included in each analysis (e.g., all randomised participants), and in which group	21-22
	21c	How missing data were handled in the analysis	21
	21d	Methods for any additional analyses (e.g., subgroup and sensitivity analyses), distinguishing prespecified from post-hoc	22
**Results**			
Participant flow, including flow diagram	22a	For each group, the numbers of participants who were randomly assigned, received intended intervention, and were analysed for the primary outcome	6,7,35
	22b	For each group, losses and exclusions after randomisation, together with reasons	6,35
Recruitment	23a	Dates defining the periods of recruitment and follow-up for outcomes of benefits and harms	6
	23b	If relevant, why the trial ended or was stopped	NA

Intervention and comparator delivery	24a	Intervention and comparator as they were actually administered (e.g., where appropriate, who delivered the intervention/comparator, how participants adhered, whether they were delivered as intended [fidelity])	6
	24b	Concomitant care received during the trial for each group	
Baseline data	25	A table showing baseline demographic and clinical characteristics for each group	29-30
Numbers analysed, outcomes and estimation	26	For each primary and secondary outcome, by group:<br>● the number of participants included in the analysis<br>● the number of participants with available data at the outcome time point<br>● result for each group, and the estimated effect size and its precision (such as 95% confidence interval)<br>● for binary outcomes, presentation of both absolute and relative effect size	6-10
Harms	27	All harms or unintended events in each group	9-10, 33-34,36
Ancillary analyses	28	Any other analyses performed, including subgroup and sensitivity analyses, distinguishing pre-specified from post-hoc	10
**Discussion**			
Interpretation	29	Interpretation consistent with results, balancing benefits and harms, and considering other relevant evidence	11-13
Limitations	30	Trial limitations, addressing sources of potential bias, imprecision, generalisability, and, if relevant, multiplicity of analyses	14

*We strongly recommend reading this statement in conjunction with the CONSORT 2025 Explanation and Elaboration and/or the CONSORT 2025 Expanded Checklist for important clarifications on all the items. We also recommend reading relevant CONSORT extensions. See www.consort-spirit.org.

Citation: Hopewell S, Chan AW, Collins GS, Hróbjartsson A, Moher D, Schulz KF, et al. CONSORT 2025 Statement: updated guideline for reporting randomised trials. BMJ. 2025; 388:e081123. https://dx.doi.org/10.1136/bmj-2024-081123.

© 2025 Hopewell et al. This is an Open Access article distributed under the terms of the Creative Commons Attribution License (https://creativecommons.org/licenses/by/4.0/), which permits unrestricted use, distribution, and reproduction in any medium, provided the original work is properly cited.
